# A Following Wave Pattern of Suicide-Related Pediatric Emergency Room Admissions during the COVID-19 Pandemic

**DOI:** 10.3390/ijerph20021619

**Published:** 2023-01-16

**Authors:** Tomer Mevorach, Gil Zur, Noa Benaroya-Milshtein, Alan Apter, Silvana Fennig, Shira Barzilay

**Affiliations:** 1 Department of Psychiatry, Feinberg Child Study Center, Schneider Children’s Medical Center of Israel, Petach Tikva 4920235, Israel; noabena@clalit.org.il (N.B.-M.); eapter@clalit.org.il (A.A.); silvanaf@clalit.org.il (S.F.); shirabarzilay@univ.haifa.ac.il (S.B.); 2 Sackler Faculty of Medicine, Tel Aviv University, Tel Aviv 6997801, Israel; 3 The Edmond and Lily Safra Center for Brain Sciences (ELSC), The Hebrew University of Jerusalem, Jerusalem 9112102, Israel; gil.zur1@mail.huji.ac.il; 4 Department of Community Mental Health, Faculty of Social Welfare and Health Sciences, University of Haifa, Haifa 3498838, Israel

**Keywords:** suicide, COVID-19, emergency room, risk assessment, suicide attempt

## Abstract

The COVID-19 pandemic and response, which included physical distancing and stay-at-home orders, disrupted the daily lives of children and adolescents, isolating them from their peers, school, and other meaningful contacts. The present study aims to add to the accumulating evidence on the pandemic’s impact on child and adolescent suicidal behavior. Data were extracted from Schneider Children’s Medical Center of Israel’s pediatric emergency room (ER) admissions for psychiatric consultation for suicidal-risk assessment between 1 January 2020, and 16 April 2022. We applied time-lagged cross-correlation analysis and a Granger causality test to assess the temporal relationships between COVID-19 infection waves and patterns of suicide-related ER admissions. The results revealed a significant lagged correlation between national COVID-19 infection rates and ER admission rates. The highest correlation was above 0.4 and was found with a lag of 80 to 100 days from infection rate to ER admission rate. The findings show that the effects of public crises change over time and may be lagged. This may have important implications for mental health services’ readiness to serve growing numbers of children and adolescents at risk for suicide.

## 1. Introduction

The COVID-19 pandemic that swept the world beginning in late 2019 was characterized by waves and troughs of infection [1,2,3]. Each wave was associated with increased public anxiety and strict measures to ensure against the spread of the disease. This involved social isolation, mask-wearing, and other stressful influences. The pandemic and response, which included physical distancing and stay-at-home orders, disrupted the daily lives of children and adolescents, isolating them from their peers, school, and other meaningful contacts. The epidemic was expected to be associated with increased mental and social distress, and psychiatric services throughout the world geared up to meet the challenge [4]. Because of the stress and social isolation, the whole spectrum of suicidal behaviors, including ideation, behaviors, and attempts, was expected to increase [5].

In the field of child and adolescent mental health, several interesting findings were published in the literature. First, several studies assessed the elevation of mental health symptomatology, treatment seeking, and psychiatric diagnoses during the pandemic [6]. Nevertheless, worsening of depression and anxiety were the most prominent phenomena in a systematic review of samples from children and adolescents around the world [7]. Among the major concerns was that the overall mental health burden, on patients, families, and caregivers, might lead to excess mortality, mainly from suicide. It was also expected that there would be a crisis of referrals for depression and suicide-like behaviors.

Over the past three years since the beginning of COVID-19, a few studies have focused on this outcome, based on government population registries. Although some researchers found an increase in fatal suicide attempts [8], this was not always clear. A large international study, using real-time official data from 21 different countries, found that in 12 countries there was a decrease in the number of suicides compared to the expected rates, and no statistically significant change in suicide rates in the rest [9]. Focusing on children’s and adolescents’ suicide deaths, Charpignon and his colleagues found a rise in mortality in 14 U.S. states [10], whereas Odd and his team did not find such a rise in youth in England [11]. An explanation for the difference in the findings in both age groups might be the timing of the research, given that other studies found that mental health measures had deteriorated by that time, as in [12,13].

Fatal suicide attempts are the extremity on a broad spectrum of expressions varying from thoughts and ideations, through behaviors and gestures, rarely reaching attempts and deaths [14,15]. These various symptoms are a major public health concern in child and adolescent psychiatry, especially in ERs and emergency care clinics. A few previous studies examined the rates of suicide-related ER admissions during the COVID-19 pandemic. These found increases in suicidal thoughts and behaviors in many regions of the globe [16]. A screening for suicide ideation and attempts in children at pediatric ERs found a significant increase in such phenomena [17]. In the United States, Yard and his colleagues found a significant rise, of 31%, in suicide-related ER admission rates compared with the rates in 2019 [13]. Ridout and her colleagues found that the incidence rates of suicide-related ER admissions were comparable in 2020 to those in 2019, except for a decrease from March to May and an increase in female admissions from June through December. Notably, the increasing trend was more prominent among those without a mental health history. The authors suggested that vulnerable groups might need further interventions and that services should adjust to and accommodate the situation [18].

Among international reports, increases in youth non-fatal suicidal behaviors during COVID-19 were also reported in France, revealing up to a 299% increase among those aged 15 years and younger in the middle of the pandemic; thus, they also report an early decrease, a finding that may reflect an irregular time-dependent pattern [19]. In Spain, up to 195% increase in suicide attempts among adolescent girls reported to happen in the peak, and 25% increase in the whole year tested. In contrast, adult suicide attempt rates were found to decrease by 16.5% in the same period of time and same data registry (Catalonia Suicide Risk Code) [20].

In Israel, two studies based on nationally representative reports of adults showed a rather different situation. One study found a decrease in suicide attempt rates under social mitigation policies [21]. Another study that collected data of patients from their health maintenance organization (HMO) also found no significant increase in 2020 compared with 2017, except for two specific months that occurred during times with no restrictions. These findings remain significant after further analyses accounting for sociodemographic groups, lockdown time intervals, and COVID-19 illness status [22].

Additionally, two studies examined the change in children’s and adolescents’ mental health. One addressed the use of ambulatory psychiatric services in the second largest HMO and revealed a significant rise in morbidity, especially among females [23]. The other explored child and adolescent admissions to the psychiatric ER and revealed interesting findings showing a continuing trend of increased admissions from the years before through the first year of the pandemic and a significant elevation above this trend in the second year [12].

Therefore, we hypothesized that there would be a considerable burden on pediatric emergency rooms for suicide-related referrals. Nevertheless, the heterogeneous findings around the world raised a question regarding the different consequences of the pandemic at the time: what may explain the substantial increase in some studies versus no change or even decrease in other studies. We therefore decided to follow the patterns of referrals to a pediatric ER over the course of the COVID-19 epidemic, intending to record the changes by the time, and further looked for temporary relationship between these two variables: incidence of coronavirus infections in Israel and suicide-related ER admissions.

## 2. Materials and Methods

### 2.1. Sample

All cases were extracted from the medical electronic records system of Schneider Children’s Medical Center in Israel. We collected all cases admitted to the pediatric ER between 1 January 2020, and 16 April 2022, that were referred for a psychiatric consultation for suicidal-risk assessment in the ER. For each case, the following data were collected: date and hour of admission, age at time of admission, date of birth, residential address, cause of referral, consultation query, and content. Cases of doubling, irrelevant consultation queries (e.g., mistaken for other units), or non-suicidal cases were excluded from the data analysis. Suicidal behaviors included ideations, suicidal threats, suicidal gestures, non-suicidal self-injury (NSSI), suicide attempts, and deliberate foreign body ingestion. This study was approved by the institutional review board of Rabin Medical Center.

### 2.2. Statistical Analysis

We ran several analyses to test the temporal relationships between COVID-19 infection waves and patterns of suicide-related ER admissions. First, the two datasets (COVID-19 infection rates and suicide-related ER referrals) were resampled for a delta of one day to allow further analysis and presentation convenience. The two datasets were presented graphically together to show the oscillatory nature of the two measures. Both time series were filtered with a uniform filter (filter size = 5). Next, we applied time-lagged cross-correlation (TLCC) analysis to both filtered datasets to detect a potential phase of interest between them. In TLCC, the correlation between the two measures was tested while moving the timeline of the COVID-19 infection rate ahead, for example, testing in what increment of time the correlation between the two measures is highest. Last, we ran a Granger causality test [24] on the two datasets.

In order to test the influence of the lockdowns, we made a *t*-test comparing the number of suicide related ER admission in the period of lockdowns to the rates in the month after, and again to the rates in similar dates three years before.

Granger causality is a statistical test applied to two time-series datasets to determine whether one series is informative in predicting the values of the other series. In order to test whether time series A “Granger-causes” time series B, we first need to select a lagged value of interest for the two series. We then fit two regression models to B, one using only the past values of B as predictors and one using both the past values of A and B as predictors. We can compare the performance of the two models using statistical tests such as the F-test or the *t*-test. If the model that includes past values of A as predictors is significantly better at predicting the future values of B than the model that includes only past values of B, then we can conclude that A “Granger-causes” B. In other words, knowing the past values of A is useful in predicting the future values of B, and the inclusion of past values of A in a model for B improves the model’s ability to make predictions.

All statistical analyses were performed using Python. For dataset preprocessing and resampling, we used the Pandas library. For filtering the datasets, we applied a uniform filter using the schipy.udimage.filters library. Granger causality was applied using the statsmodels.tsa.stattools library.

## 3. Results

We collected 1091 cases of psychiatric consultations for suicide-risk assessment in the pediatric ER from electronic medical records for the 26-month period studied. Excluding cases that did not meet the study’s inclusion criteria and removing false records yielded 846 cases for data analysis. Descriptive statistics for the included cases are presented in Table 1.

**Figure 1:**
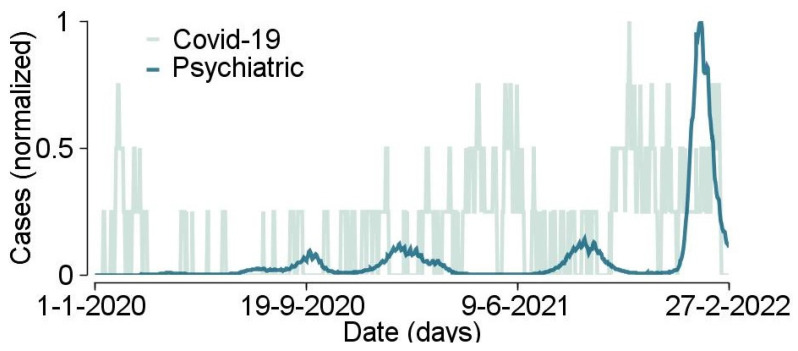
COVID-19 infection rates and suicide-related emergency room admissions in Israel, 1 January 2020, through 27 February 2022.

The prevalence of normalized COVID-19 infection rates and suicide-related ER admissions rates is shown in Figure 1. The COVID-19 infection rates have a strong and clear oscillatory nature, a well-known phenomenon of infectious “waves” published in previous studies. The ER suicide behavior related admissions, by contrast, did not show a clear oscillatory nature of prominent and distinctive periods of elevation and depression.

**Figure 2:**
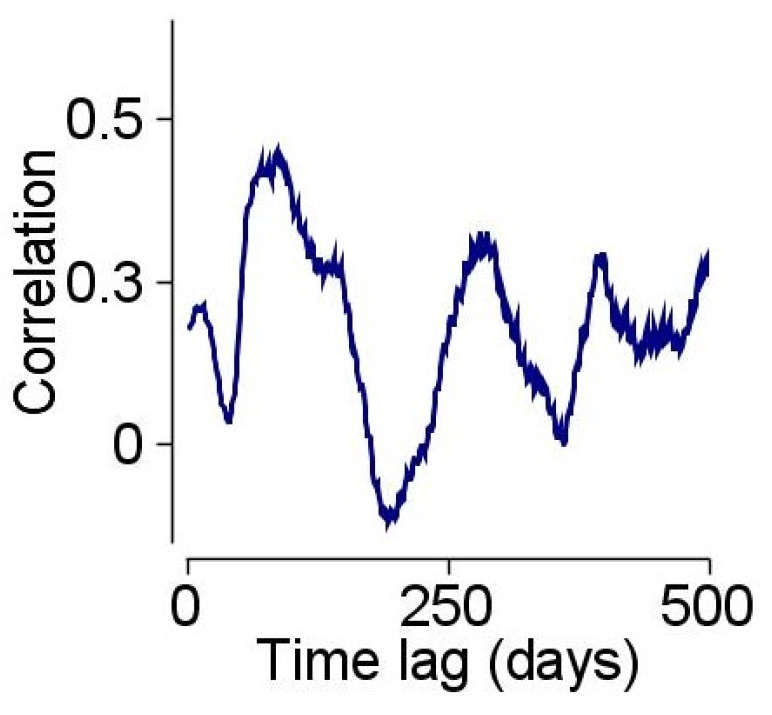
Time-lagged cross-correlation analysis of both filtered datasets, showing correlation rates by the phase of interval in timelines.

TLCC revealed that the highest correlation between the two measures, which was above 0.4, was found with a lag of 80 to 100 days. The exact correlation peak is subject to minor changes manipulated by the chosen filter size. However, the prolonged positive correlation wave is characterized by all filter-size values. The correlation was clearly lower for any other time lag before or after this period (Figure 2). The correlation graph is also oscillatory, reflecting the relationships between the previous infection “waves” and future patterns of suicide-assessment rates in the ER. A *t*-test comparing ER suicide-related admissions during lockdowns and the month after found no significant difference, *p* > 0.1 for all three lockdowns. The comparison to the rates of ER suicide-related admissions for similar dates in 2017 found a significant difference only for the third lockdown (*p* = 0.008), but the significance was lost after correction for multiple comparisons.

**Figure 3:**
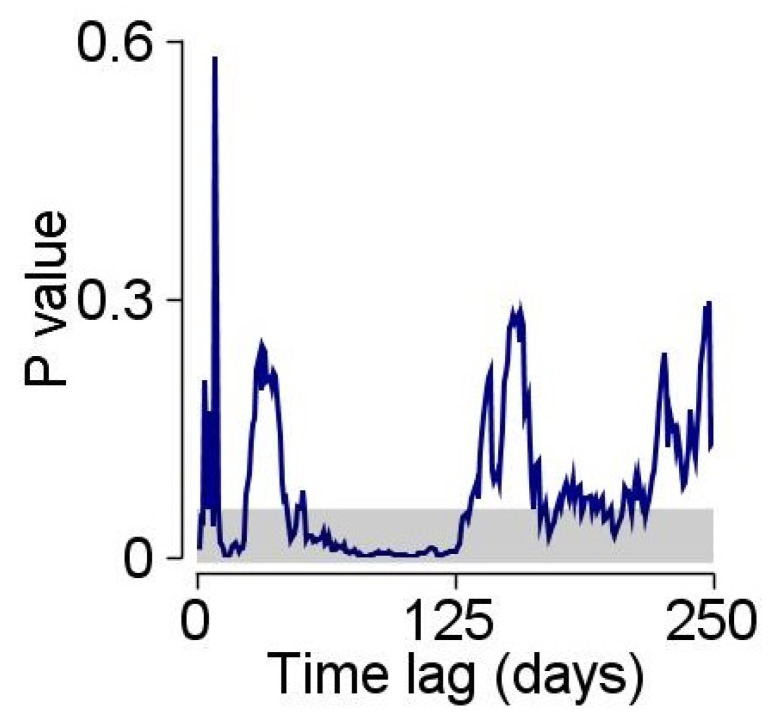
Granger causality test applied to determine for which time lag the data on COVID-19 infection rates is most predictive of suicide-related ER admissions. The gray area indicates the zone where the predictability was statistically significant.

Granger causality analysis [24] also showed significant prediction rates for the same time lag found in the cross-correlation analysis (Figure 3). The *p*-value was significant (<0.05) between days 75 and 100, indicating that for this time lag, the prediction probability from infection rate to risk assessment rate is significant.

## 4. Discussion

The aim of the present study was to examine the patterns of admissions to a pediatric ER over the course of the COVID-19 epidemic. The most striking result of our observation is the time lag between the peak of COVID-19 incidence and the peak of suicide-related ER admissions. Whereas previous reports suggested either an increase in ER suicide-related admissions during the COVID-19 pandemic (e.g., [13,19,20]) or no significant change [18,21,22], our findings emphasize the lagged response. We had expected these two phenomena to be directly related, but the finding of a time lag was unexpected. In considering the lagged increase in ER admissions, several explanations may be relevant: one might be that during COVID-19 exacerbations, children and adolescents did not attend school and stayed at home. The scholastic and social demands were greatly reduced. As the COVID-19 peak decreased and children returned to school, pressure increased with the added complications of having to make up for the lost time. Although education authorities made great efforts to institute remote learning, this was not always easy to do. In addition, during the extended stay at home, children faced other distractions, such as computer games, that masked their feelings and needs [25,26,27].

Yet another interesting comparison is with the suicide statistics for the 20th century overall, where there is a continuous increase in suicide with two prominent exceptions: during World War I and World War II [28]. This is usually explained by the fact that during the wars, young males who are at the highest risk for suicide stayed together with their comrades fighting for a common purpose. This might be a protective factor that is not relevant in the case of a pandemic. However, one might suggest that a pandemic, just like wars, unites the population against a sudden, unexpected threat that changes their whole life’s routines. In such a period, inner needs and impulses are repressed and silenced. People may cope by facing the challenge or may feel that they belong to the group of all those facing the same challenge, a concept termed “pulling together” by Joiner et al. [29]. Children’s and adolescents’ suicidal behaviors are highly related to interpersonal problems, including rejection and conflict, particularly within the family [30]. Thus, a sense of belonging and family “pulling together” may be a strong protective factor during infection waves.

Furthermore, it is possible that young people avoided coming to the ER for fear of being infected with COVID-19 and that only when infection waves decreased did they feel comfortable doing so [31].

However, the long-term impact of COVID-19 on the mental health of children and adolescents, and particularly on suicidal behavior, remains unclear. We may see the lag effects of reduced educational and social support for children during the pandemic. The current study highlights the increase in child and adolescent crises services shortly following the COVID-19 infection waves, as manifested by increased suicide-related ER visits. There are some important public health implications of our results. In most countries of the Western world, the prevailing concept was that there was an increased need for services during the pandemic, especially when lockdowns were most severe [32]. However, it may well be that the increased efforts to provide mental health services should paradoxically occur when the pandemic fades and children go back to school [33]. Thus, in our own situation, we were able to manage during the pandemic despite the fact that we were short staffed. Now that the pandemic is over and there is less pressure for an increase in mental health services from governmental stakeholders, we have found ourselves overwhelmed with increasing referrals and ever-lengthening waiting lists.

Future studies should examine the long-term effects of direct preventive measures by public health systems, including shifts in service-delivery methods, as mental health needs evolve.

## 5. Limitations

Our study has a few methodological limitations. First, it includes data from one of the biggest children’s hospitals in the Middle East, but it is only a single medical center. Different patterns may be seen when analyzing nationwide or international multi-center data. Moreover, as a retrospective study, it might have some more confounders given that other factors may have also influenced the rates of suicide-related pediatric ER admissions. We addressed some of the potential confounders in the discussion, but there might be more options. Although COVID-19 infection rates and their effect on suicidality may vary across age groups, genders, and residences [4,6], this study did not account for such differences as they were determined to be beyond the scope of the current study. Finally, in this work we did not make statistical tests in order to compare the ER suicide-related admission rates in the research period to the rates of another year’s ER suicide-related admissions; thus, we cannot prove that the findings are unique to the study period.

## 6. Conclusions

This study adds to the growing body of knowledge about mental health and, in particular, suicidal behavior trends during the COVID-19 epidemic. It shows that there is a lagged response, with an increase in ER admissions due to suicidal behaviors about three months following COVID-19 infection waves.

Previous research on mental health during the pandemic has largely focused on comparing the times of a pandemic with normal times, but our study takes a different approach and found evidence of temporary changes during the pandemic itself. Specifically, we identified a unique oscillatory pattern in emergency room admissions due to suicidal risk during the COVID-19 pandemic. These changes were found to be correlated with the waves of COVID infections, which raises the possibility of causality between the two. This finding requires further exploration to understand the nature of this connection and its potential implications, as well as to identify potential interventions to address the increase in suicidal emergency department admissions during pandemics.

Our results show that the effect of public crises changes over time and may be lagged. These are important insights for preventing suicide and suicidal behaviors in children and adolescents. Our findings suggest that public health efforts and suicide prevention strategies should focus not on the times of the pandemic peak but rather on a few months later, the times when the emergency situation fades away and children and adolescents have to get back to the routines of their lives.

Overall, our study offers a new perspective on pandemics and highlights the need for ongoing research to fully understand their impact.

## Figures and Tables

**Table 1:** Descriptive statistics of the sample characteristics.

	Percentage	N
Total	100%	846
Males	37%	310
Females	63%	536
Daytime (8–16)	60%	511
Evening (16–00)	32%	271
Nighttime (00–08)	8%	64
Prepubertal	25%	215
Adolescents (12–18)	75%	631
Summer (21 June–20 September)	16%	121
Winter (21 December–20 March)	25%	192
Autumn (21 September–20 December)	32%	239
Spring (21 March–20 June)	27%	204

## Data Availability

Not applicable because of the privacy policy of the IRB.
